# A Review of Multi-Robot Systems and Soft Robotics: Challenges and Opportunities

**DOI:** 10.3390/s25051353

**Published:** 2025-02-22

**Authors:** Juan C. Tejada, Alejandro Toro-Ossaba, Alexandro López-Gonzalez, Eduardo G. Hernandez-Martinez, Daniel Sanin-Villa

**Affiliations:** 1 Departament of Engineering Studies for Innovation, Universidad Iberoamericana Ciudad de México, Prolongación Paseo de la Reforma 880, Colonia Lomas de Santa Fé, Ciudad de México 01219, Mexico; juan.tejada@ibero.mx (J.C.T.); alexandro.lopez@ibero.mx (A.L.-G.); 2 Artificial Intelligence and Robotics Research Group (IAR), Universidad EIA, Envigado 055428, Colombia; alejandro.toro52@eia.edu.co; 3 InIAT Institute of Applied Research and Technology, Universidad Iberoamericana Ciudad de México, Prolongación Paseo de la Reforma 880, Colonia Lomas de Santa Fé, Ciudad de México 01219, Mexico; eduardo.gamaliel@ibero.mx; 4 School of Applied Sciences and Engineering, Universidad EAFIT, Medellín 050022, Colombia

**Keywords:** multi-agent systems, multi-robot systems, soft robotics, collaborative robotics, systems integration

## Abstract

This review investigates the latest advancements in Multi-Robot Systems (MRSs) and soft robotics, with a particular focus on their integration and emerging opportunities. An MRS extends principles from distributed artificial intelligence and coordination frameworks, enabling efficient collaboration in robotic applications such as object manipulation, navigation, and transportation. Soft robotics employs flexible materials and biomimetic designs to improve adaptability in unstructured environments, with applications in manufacturing, sensing, actuation, and modeling. Unlike previous reviews, which often address these fields independently, this work emphasizes their integration, identifying key challenges such as nonlinear dynamics, hyper-redundant configurations, and adaptive control. This review discusses recent advancements in locomotion, coordination, and simulation, offering insights into the development of adaptive and collaborative robotic systems across diverse applications.

## 1. Introduction

Multi-Agent Systems (MASs) are a branch of artificial intelligence, particularly distributed artificial intelligence, aimed at solving problems with a distributed nature by coordinating multiple agents. The core of MASs lies in their ability to address tasks that are difficult or impossible for a single agent to handle independently [1,2]. A specific subfield that applies the multi-agent paradigm to robotics is MRSs. An MRS is formally defined as a group of robots organized based on a multi-agent architecture, allowing them to work collaboratively toward solving either a single task or multiple tasks [3].

According to the literature, various authors have proposed different classifications for MRSs [3,4]. Verma and Ranga [3] categorize MRSs into five dimensions, depending on how agents interact, their composition, communication mechanisms, and other factors.

The first classification dimension pertains to the coordination type within the MRS. Coordination refers to the mechanism used to achieve cooperation among the agents in the system. Coordination mechanisms can be classified as strong or weak [4], depending on the level of cooperation required. Furthermore, coordination can be centralized or decentralized, based on how decisions are made within the system. In a centralized architecture, all agents share knowledge about each other, and decisions are made by a central computer or specific agent. In contrast, a decentralized architecture allows each agent to make decisions independently, offering greater flexibility [5]. In previous classifications, it is assumed that agents are aware of others in the system; however, MRSs can still achieve coordination even when agents have no knowledge of other agents. Such systems are referred to as uncoordinated MRSs [6]. While uncoordinated MRSs offer simpler designs, they are often less efficient as agents may conflict while performing similar tasks.

The second classification dimension focuses on the composition or types of agents. Based on this, an MRS can be homogeneous or heterogeneous. Homogeneous MRSs consist of robots with identical hardware and software, whereas heterogeneous MRSs involve robots that differ in software, hardware, or both. In recent years, research on heterogeneous MRSs has increased due to their greater versatility in performing diverse tasks, although this comes with added complexity in system coordination [7].

The third classification pertains to the type of cooperation within the MRS. Cooperative MRSs are systems in which all agents or robots work together to achieve a specific objective. On the other hand, competitive MRSs consist of agents acting independently and competing to fulfill individual objectives [3].

The fourth classification dimension relates to how agents communicate. MRSs can operate with communication mechanisms that enable agents to share information. As previously mentioned, if communication is absent, the MRS is considered uncoordinated. Communication among agents can be explicit or implicit. Explicit communication involves dedicated hardware that allows agents to directly exchange signals, while implicit communication occurs when agents gather information about others through environmental interactions. Yan et al. [8] distinguish implicit communication mechanisms as passive (using sensors) or active (through interactions with the environment).

The fifth and final classification considers how robots in an MRS adapt to their environment, which can be either reactive or deliberative. Deliberative MRSs adapt to environmental changes through approaches that restructure the overall behaviors of the team. In contrast, reactive MRSs involve each robot individually adapting to changes in the environment, reorganizing its behavior to successfully complete the assigned task.

**Figure 1:**
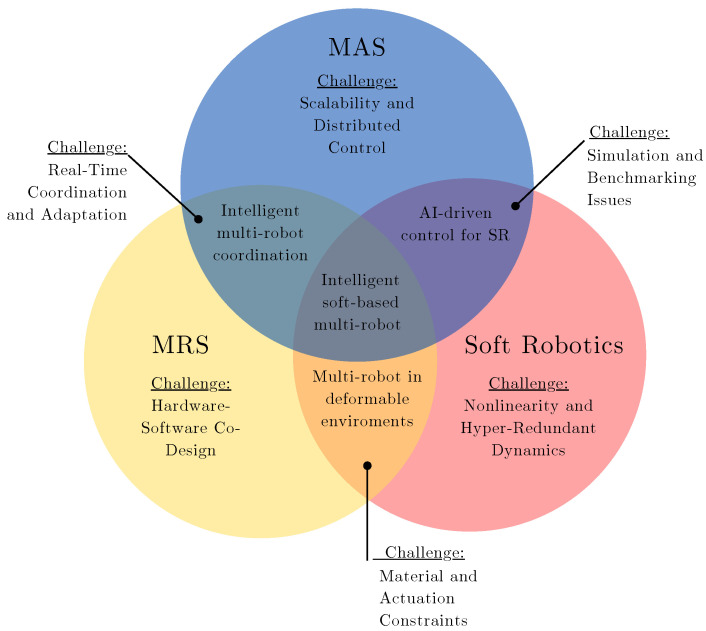
Visualization of the relationship between Multi-Agent Systems (MASs), Multi-Robot Systems (MRSs), and soft robotics.

One of the current applications of MRSs is coordinated object manipulation, where multiple agents collaborate to move an object from one point to another [9]. The advantage of employing an MRS is the ability to handle large and/or heavy objects that cannot be transported by a single robot [4]. Current research focuses on developing control strategies that enable effective MRS coordination during object manipulation [4,10,11]. A crucial factor in these control strategies is the design of the contact interface or the gripping method used by each robot to hold or move the object. With the growing interest in soft robotics—an emerging field that develops soft interfaces or actuators for object manipulation—new challenges have arisen in designing control schemes that integrate MRS coordination with the interaction of each agent with the object. As shown in Figure 1, the integration of MASs, MRSs, and soft robotics presents several challenges and opportunities. The overlapping areas highlight key research directions such as scalability, adaptive AI-based control, and collaborative multi-robot interactions in unstructured environments.

To provide a structured analysis, this review is organized as follows: Section 2 presents the principles and classifications of MASs and MRSs, highlighting their key characteristics, coordination mechanisms, and applications. Section 3 focuses on the field of soft robotics, discussing its fundamental concepts, material properties, and advances in fabrication, sensing, and actuation. Section 4 explores the integration of MASs/MRSs with soft robotics, emphasizing collaborative object manipulation, adaptive control strategies, and communication frameworks. Section 5 identifies the main challenges and opportunities in merging these fields, proposing future research directions to overcome current limitations. Finally, Section 6 concludes with a summary of key findings and the potential transformative impact of integrating MRSs and soft robotics on dynamic, real-world applications.

## 2. Methodological Approach

This review investigates the integration of MASs, MRSs, and soft robotics, focusing on their combined challenges and opportunities. To ensure comprehensive coverage of the latest advancements, a systematic literature search was conducted using the SCOPUS and Web of Science databases, with additional support from tools such as Research Rabbit and Elicit for data exploration and synthesis. These tools facilitated iterative searches, relationship mapping, and identification of relevant studies in this emerging interdisciplinary field.

### 2.1. Search Strategy

The search employed structured queries combining Boolean operators and keywords aligned with the main topics of MASs, MRSs, and soft robotics. Keywords were grouped into three themes:

- MASs and MRSs: “multi-agent systems”, “multi-robot systems”, “autonomous coordination”, “collaborative robotics”.
- Soft Robotics: “soft robotics”, “soft actuators”, “soft sensors”, “biomimetic robotics”.
- Integration and Applications: “coordination in soft robotics”, “adaptive control”, “collaborative manipulation”, “simulation of hyper-redundant dynamics”.

An example query used in SCOPUS was as follows:

TITLE-ABS-KEY(("multi-agent systems" OR "multi-robot systems"

OR "collaborative robotics") AND

("soft robotics" OR "soft actuators" OR "soft sensors") AND

("coordination" OR "adaptive control" OR "simulation"))

### 2.2. Inclusion and Exclusion Criteria

The selection process targeted peer-reviewed articles based on their relevance and significance in the fields of Multi-Agent Systems, Multi-Robot Systems, and soft robotics, regardless of their publication year. The inclusion criteria were as follows:

- Research addressing the integration of MASs, MRSs, and soft robotics.
- Studies exploring synergies or challenges at the intersection of these fields.
- Articles providing experimental, theoretical, or simulation-based insights.

Articles were excluded for the following reasons:

- They did not address soft robotics, MASs, or MRSs.
- They focused exclusively on rigid robotic systems.
- They lacked full-text access or were not peer-reviewed.

### 2.3. Data Filtering and Selection

The initial search identified approximately 800 articles. After removing duplicates and screening the abstracts for relevance, 323 articles were subjected to full-text review. A final dataset of 143 articles was selected, emphasizing works that addressed soft robotics and key integration challenges, such as nonlinear dynamics, adaptive control, and coordination in unstructured environments.

**Figure 2:**
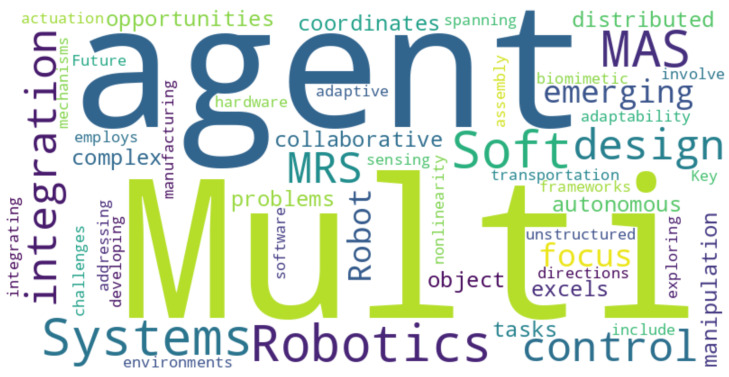
Word cloud summarizing the key themes and concepts explored in this review, highlighting the integration of Multi-Agent Systems, Multi-Robot Systems, and soft robotics.

A word cloud was generated based on the extracted keywords and core ideas from the analyzed literature to provide a visual summary of the key themes and concepts explored in this review. This visualization, presented in Figure 2, highlights the prominence of topics such as “Multi-Agent Systems”, “Soft Robotics”, “integration”, “control”, and “collaborative tasks”, reflecting the interdisciplinary nature and focus of the study. By emphasizing the frequency of these terms, the word cloud serves as a concise representation of the research landscape, offering insights into the main challenges, opportunities, and directions identified throughout the review.

## 3. Cooperative Object Transportation

The manipulation of objects by mobile robots, particularly within Multi-Robot Systems, has been extensively studied in recent years due to the unique capabilities of these systems to transport very heavy, large, or geometrically complex objects that would be impossible for a single robot to handle. This capability is particularly valuable for applications with significant economic and social impact, such as waste recovery and disposal, mine clearance, or operations that require handling objects in environments where direct human intervention is impractical or unsafe [12].

**Figure 3:**
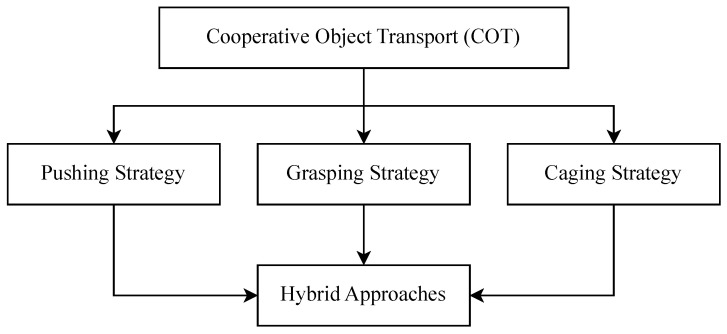
Cooperative Object Transport (COT) manipulation strategies.

While multiple robots are often used for these tasks, it is not necessary for all robots in the system to handle the object directly. Cooperation within the system can still occur when some robots manipulate the object while others coordinate and plan the trajectory or handle obstacle avoidance [13]. Three primary object manipulation strategies have been widely studied in the literature: pushing, grasping, and caging [12]. For maritime applications, additional strategies, such as towing, have also been explored [14]. A summary of these strategies can be seen in Figure 3.

The pushing strategy involves robots moving the object by pushing it without necessarily being physically attached to or in constant contact with the object [15]. Several studies have focused on pushing strategies, including developing three submersible robots that decompose the task into subtasks and communicate using a market-based dynamic task allocation method, where two robots push the object while the third plans the trajectory [16]. Other notable works include the design of an automatic feedback linearization controller to push an object using a single autonomous floating vehicle [17], the development of behavior-based controllers for robot groups [18], and a pushing strategy based on occlusion, where robots push the object toward areas where the object itself blocks their line of sight [19].

The grasping strategy involves robots physically attaching to the object to push, pull, or perform both actions. Noteworthy studies in this area include developing a telescopic grasping system that uses a mesh to hold objects of different geometries and can be mounted on any type of aerial autonomous robot [20]. Additional work has focused on decentralized sliding mode controllers for object transportation using robot groups [21], a decentralized force-feedback controller for coordinating contact forces between robots and the object without requiring inter-robot communication [22], and a distributed force and torque controller that enables both rotational and translational movements of the object [23].

The caging strategy involves robots surrounding and “trapping” the object to hold it securely during transportation. This approach has been extensively studied due to the coordination required among multiple robots to enclose the object. Examples include the development of a group of robots capable of enclosing a cluster of agents and guiding them to another location without escape, using a rapid random tree (RRT)-based path planning algorithm [24]. Other contributions include an adaptable model predictive controller (MPC) designed to move a heavy body on a high-friction surface by loosely enclosing the object to minimize the required torques [25], a decentralized algorithm that allows the transportation of arbitrarily shaped objects without prior knowledge of the object’s geometry [10], and a fuzzy sliding mode controller that prevents the object from escaping the grip [26]. Research has also focused on robust caging strategies that minimize the number of robots required to grip the object [27], analyses of the difficulty of transport operations based on the geometry and mechanical constraints of the robots and the object [28], and convex combinatorial models for optimizing caging operations in mobile robot object manipulation tasks [29].

In addition to these strategies, other innovative approaches have been proposed. One example involves a swinging strategy where the robot pivots the object side-to-side on a fulcrum, causing it to move in a zigzag pattern [30]. Another approach utilizes deep reinforcement learning techniques to enable robots to learn how to transport the object [31]. Other authors have focused on the development of digital twin models that allow an MRS to handle complex-shaped parts in an assembly line by learning from a remote operator using a virtual simulation environment [32]. Garg et al. reviewed control design methods for MRSs focusing on learning-based control methods with safety considerations such as shielding-based methods, methods for learning control barrier functions (CBFs), reinforcement learning methods, and safety verifications of the control policy [33].

## 4. Theoretical Framework and State of the Art in Soft Robotics

Soft robots, characterized by their flexible structures and redundant degrees of freedom, are particularly suited for delicate tasks in unknown and unstructured environments, offering exceptional dexterity. These systems exhibit distributed deformation, theoretically possessing infinite degrees of freedom. This results in hyper-redundant spatial configurations where the robot’s end-effector can reach any point within the three-dimensional workspace through infinite possible shapes. Unlike hyper-redundant rigid robots, soft robots have the added advantage of generating minimal resistance to compressive forces, allowing for superior obstacle negotiation capabilities [34].

Soft robots primarily comprise easily deformable materials such as fluids, gels, and elastomers, which provide the structural flexibility needed for their operation. When two materials interact, they must share similar mechanical stiffness to distribute internal loads and minimize surface stress concentrations evenly [35]. This principle, however, does not apply to interactions between rigid robots (with a Young’s modulus exceeding $E=10^{9}\;\mathrm{Pa}$) and soft materials (ranging from $E=10^{2}$ to $10^{6}\;\mathrm{Pa}$), which can lead to material damage. Such interactions are common in scenarios involving human tissue, skin, delicate organs, or biological entities. Due to this mechanical mismatch, rigid robots are not well suited and can even be hazardous for human interaction. Consequently, a growing need for robots matching the elastic and rheological properties of natural materials and organisms is growing, making soft robotics a promising solution.

Several authors have proposed definitions of soft robotics to capture its essence. Wang and Iida define soft robotics as the study of how the deformation of soft materials can be utilized or controlled to achieve robotic actions [36]. Laschi and Cianchetti extend the concept beyond material-based definitions, describing soft robotics from two perspectives: controlling the stiffness of actuators in robots with rigid links and achieving intrinsic softness through passive characteristics of the robot’s coating or structure [37]. Similarly, Chen and colleagues define softness in robotics as the stress and other forces generated in a robot’s environment due to specific material deformations and structural configurations, further stating that soft robotics involves using such softness to build robots that meet the softness requirements of their environments and themselves [38].

In 2016, the RoboSoft community defined soft robots as flexible devices capable of actively interacting with their surroundings while undergoing “large” deformations due to their inherent or structural flexibility. For further exploration of definitions in contexts such as soft structures, soft control, and other behaviors, the work of Wang, Nurzaman, and Iida provides valuable insights [39].

### 4.1. Fabrication

Using soft materials in robot construction introduces challenges in many robot functions. Working primarily with elastic and nonlinearly deformable materials increases the complexity of tasks such as transmission, sensing, and actuation, requiring a more thorough and carefully planned design phase [40]. For this reason, fabrication methods in this field need to be highly structured and tailored to the specific requirements of soft robotics, more so than in other applications. However, as this technology is still in its early stages, researchers have developed diverse fabrication methodologies that yield vastly different results, which are not necessarily inferior but reflect the experimental nature of this field. Most studies rely on finite element analysis to predict the various behaviors of materials under deformation [40,41].

In soft robotics, most studies to date have focused on silicone elastomers [40]. Consequently, the most common fabrication technique for soft robots is molding or lithography-based molding, which involves creating molds using 3D printing or lithography to produce physical forms from elastomers that make up the soft robot’s structure [38,42]. This process typically consists of three main stages: designing and fabricating the molds, using these molds to create internal and external parts or components with different stiffnesses, allowing for the curing of the parts, and, if necessary, demolding. Finally, the parts are joined using the same elastomer as an adhesive [40,43].

Table 1 outlines various fabrication methods used for soft robotic components.

### 4.2. Materials

A robot is considered soft based on the compressibility of the materials that compose it [34]. The body of a soft robot can be formed from various materials with different hardness properties [44,45]. An autonomous soft robot integrates all the subsystems of a conventional robot into a soft body, including an actuation system, a perception system, power electronics, and a computational system with its corresponding energy source. Advances in materials and subsystems compatible with soft bodies have enabled the autonomous functionality of soft robots.

The following is a brief overview of materials found in the literature that have been used in soft robotics applications. These materials are categorized into four groups [46].

Hydrogels are polymers with unique properties, including excellent physicochemical characteristics, high expandability, self-regeneration, biocompatibility, and exceptional electrical conductivity. These properties make hydrogels highly adaptable to the requirements of soft robotics [47,48].

Liquid metals, with their intrinsic properties of conductivity and deformability, offer an intriguing option for soft robot design. However, some liquid metals, such as francium, cesium, and rubidium, are radioactive or highly reactive when exposed to air. Magnesium, on the other hand, has been utilized in innovative ways within the electrical industry [49,50].

Conductive polymers are composed of long polymer chains that provide inherent flexibility and durability. These polymers are modified through doping processes that introduce electrical conductors along the chains, giving them conductivity [51,52].

Nanomaterials encompass numerous subclasses defined by their fabrication methods. These materials typically involve bottom-up manufacturing processes, granting them tailor-made characteristics, such as desired mechanical properties, flexibility, hardness, and conductivity, among others [53,54].

Table 2 lists the principal materials used in soft robotics, highlighting their properties and applications.

### 4.3. Actuators

The sensing and control of soft robots present significant challenges, as the entire robot often acts as the actuator. Inspired by the remarkable capabilities of soft animals and plant structures, researchers have developed both rigid robots that mimic soft structures and soft robots that employ actuators such as electroactive polymers (EAPs) and pneumatic artificial muscles (PAMs). The use of these materials in robotics is not entirely new, as many rigid-structured robots rely on electrical/magnetic, piezoelectric, or thermal actuation systems, such as shape memory alloys (SMAs). Currently, various robotic manipulators have been built using rigid structures and electric motors that drive cables simulating tendons for actuation [34,55,56].

#### 4.3.1. Thermal Actuators

Thermal actuators include silicone–ethanol devices that utilize ethanol embedded in silicone elastomers. These actuators expand due to ethanol vaporization upon heating, producing volumetric changes. While they are cost-effective and easy to fabricate, uneven heating caused by wire elements can result in slower and non-uniform actuation. Heat-conducting fabrics have been proposed to address these limitations [57].

#### 4.3.2. Pneumatic Actuators

Pneumatic actuators are widely used in soft robotics. These actuators include pneumatic muscle actuators (PAMs) and elastomeric chambers reinforced with fibers, which produce bending, twisting, or extension trajectories when pressurized. PAMs mimic the functionality of biological muscles and have been applied in industrial and medical contexts due to their low cost and high force-to-weight ratio [56]. Elastomeric pneumatic actuators are often fabricated using materials such as Elastosil M4601 and Wacker Chemie AG, enabling precise motion programming for applications like finger actuation [58,59,60].

#### 4.3.3. Electroactive Polymer (EAP)

Electroactive polymers (EAPs) are highly advantageous for soft robotics due to their light weight, flexibility, high fracture resistance, and large actuation strain. EAPs are categorized into electronic EAPs and ionic EAPs. Electronic EAP include dielectric elastomers, liquid crystal elastomers, and ferroelectric polymers, which offer high actuation forces, rapid responses, and energy efficiency but require high voltages. Conversely, ionic EAPs, such as ionic polymer–metal composites (IPMCs) and carbon nanotubes, operate at low voltages but produce lower forces and require constant hydration [61].

Dielectric elastomer actuators (DEAs), a type of electronic EAPs, are widely employed in soft robotics for their large deformation capabilities and fast responses. DEAs consist of a dielectric elastomer membrane sandwiched between two compliant electrodes, compressing under an applied electric field [62].

#### 4.3.4. Light-Driven Actuators

Light-driven actuators leverage the precision of light stimuli for spatial and temporal control. For example, light-responsive gels contract under UV irradiation and revert to their original state under visible light. These actuators provide reversible deformation, making them suitable for precise robotic applications [63].

#### 4.3.5. Pouch Motors

Pouch motors use inflatable “pouches” that deform upon fluid pressurization, producing linear or rotational movements depending on the pouch design. By combining multiple pouch motors, soft robots can achieve complex tasks, making them versatile components in soft robotics [64].

#### 4.3.6. Magnetic Actuators

Magnetic actuators, including magnetorheological fluids (MRFs) and magnetorheological elastomers (MREs), utilize magnetic fields to alter their properties. MRFs consist of ferromagnetic particles suspended in a fluid medium, whereas MREs combine polymers like silicone with ferromagnetic materials. While MREs address contamination and sedimentation issues seen in MRFs, both systems enable precise actuation under magnetic fields [65].

#### 4.3.7. Compliant Mechanism Actuators

Compliant mechanisms achieve mobility through structural deformation rather than rigid joints. They are categorized into concentrated and distributed compliance based on the scale of deformation. Compliant mechanisms offer advantages such as complex motion trajectories and reduced reliance on sensory systems. Examples include compliant grippers optimized for specific tasks and fluid valves driven by internal pressure. Despite their advantages, the mathematical modeling of compliant mechanisms remains challenging due to their nonlinear behavior [66].

#### 4.3.8. Applications and Integration

The integration of various actuation technologies has led to significant advancements in soft robotics. Soft manipulators and grippers utilizing pneumatic actuators, EAPs, and compliant mechanisms have demonstrated their potential in tasks requiring adaptability, dexterity, and precision. By combining these technologies, researchers continue to push the boundaries of robotic capabilities, bridging the gap between biological and engineered systems [34,55,56]. Table 3 presents the most widely used actuation mechanisms in soft robotics.

### 4.4. Soft Sensors

Soft robotic manipulators enable tasks that traditional rigid robots cannot achieve. However, creating soft actuators with multiple degrees of freedom and suitable sensory capabilities remains a significant challenge. To address these needs, various types of soft sensors have been developed, each utilizing different mechanisms and fabrication techniques.

#### 4.4.1. Inductive Sensors

Inductive sensors operate by altering inductance due to deformation. A notable example is a sensor that uses liquid metal encased in silicone, connected to a coil circuit. When the sensor bends, the liquid metal changes the inductance, producing an electrical signal [67].

#### 4.4.2. Resistive Sensors

Resistive sensors detect changes in resistance caused by deformation or applied forces. These sensors come in various designs, including simple sensors based on ferromagnetic powder [68] or carbon black encapsulated in silicone for low-cost and straightforward fabrication [69]. Advanced designs include 3D-printed sensors made of thermoplastic polyurethane [70] and microchannels filled with liquid metals like Galinstano or eutectic gallium–indium (eGaIn) [71,72]. Hybrid sensors capable of measuring both pressure and elongation [73], as well as resistive thermal sensors combining carbon nanotubes with polymers [74], have also been proposed. Furthermore, artificial skins using ionized water channels have demonstrated high precision in force localization [75]. These resistive sensors have broad applications, including haptic feedback, wearable devices, and robotic skins.

#### 4.4.3. Capacitive Sensors

Capacitive sensors measure changes in capacitance due to deformation or external forces. Examples include sensors inspired by Merkel cells, which use PDMS molded to reduce cross-coupling [76], and DIY designs using PDMS and aluminum layers for simple capacitor construction [77]. Additionally, porous structures with micro-nanopores have been developed to enhance sensitivity [78], and multifunctional sensors capable of detecting pressure, deformation, and shear forces have been demonstrated [79].

#### 4.4.4. Optical Sensors

Optical sensors provide high-resolution tactile feedback using light-based mechanisms. Vision-based sensors paired with neural networks predict force distribution with high precision [80]. Others integrate optical components with pneumatic structures to measure pressure and deformation [81], or use tactile optical systems employing LEDs and photodiodes embedded in soft materials [82].

#### 4.4.5. Magnetic Sensors

Magnetic sensors rely on composites such as silicone or urethane foam infused with magnetic particles. These sensors detect changes in the magnetic field to determine force and location, though they can be sensitive to environmental noise [83].

#### 4.4.6. Hybrid Sensors

Hybrid sensors combine multiple principles to enhance versatility. Examples include sensors with wrinkled surfaces that improve texture and shape detection [84], as well as bubble arrays utilizing carbon black membranes to measure both pressure and deformation simultaneously [85].

#### 4.4.7. Sensor–Actuator Integration

Some designs integrate sensing and actuation within the same system. Ionic polymer–metal composites (IPMCs) function as both sensors and actuators by utilizing ion migration when subjected to voltage [86]. Pneumatic actuators paired with resistive or optical sensors enable closed-loop control and enhanced functionality [87,88].

#### 4.4.8. Applications of Soft Sensors

The applications of soft sensors are diverse and impactful. For instance, soft grippers equipped with pressure and tactile sensors allow precise object manipulation [89]. Robotic feet embedded with tactile, acoustic, capacitive, and thermal sensors have been employed for terrain classification, leveraging machine learning algorithms like KNN, SVM, and Random Forest [90]. These examples demonstrate the transformative potential of soft sensors in enabling adaptive, dexterous, and intelligent robotic systems. Table 4 categorizes the primary types of sensors used in soft robotics, highlighting their functions and applications.

### 4.5. Deformable Electronics

To date, most soft robot designs rely on conventional rigid electronics, either placed externally or embedded within the robot’s soft structure. However, in recent years, significant advancements have been made in the field of soft and deformable electronics, as highlighted in [91,92,93,94]. These developments have propelled the field of soft robotics, enabling new levels of autonomy and expanding the potential applications of soft robotic systems. The integration of deformable electronics has allowed researchers to address challenges associated with embedding electronics into highly deformable and dynamic structures, thereby improving the functionality and versatility of soft robots.

### 4.6. Control of Soft Robots

Soft robots face numerous challenges in motion control, primarily due to their hyper-redundant degrees of freedom. To address this, control strategies or models must be developed to reduce the complexity associated with their degrees of freedom through coordination. This issue is a critical problem in the motion control of such robotic systems [61].

Soft robots are categorized based on their fabrication materials or actuation systems, and it is essential to establish control systems that align with the specific technology employed. Generally, model-based controllers have been implemented for systems with defined degrees of freedom or linearized systems, enabling highly precise responses. In contrast, unstructured applications—such as atypical geometries, hyper-redundant degrees of freedom, or systems that cannot be linearized—often require model-free control systems [95]. Dynamic controllers, commonly used in cable-actuated manipulators, are typically absent in soft robotic systems. This absence is attributed to the difficulty of establishing uniform loads on tendons, which would enable the use of linear models [95]. In some cases, sliding mode control (SMC) has been implemented. One advantage of SMC is that it does not require an explicit system model for controller synthesis, provided the system behavior is continuous and sufficiently smooth [60].

These challenges have driven the development of intelligent control strategies. Researchers have explored the implementation of autonomous systems capable of learning from their environment to execute predefined tasks. This approach leverages deep learning techniques, including imitation learning agents, which address the difficulty of defining appropriate control rules [96]. Despite these advances, some control applications have successfully employed feedback-based control systems for pneumatic soft manipulators. These implementations use flexible load sensors and actuators such as Flexible Fluidic Actuators (FFAs) [97].

Table 5 classifies the primary control approaches used in soft robotics.

### 4.7. Mathematical Models

Soft robotic systems exhibit unique characteristics when it comes to modeling, primarily due to their nonlinear kinematic behavior. The motion of these highly deformable systems is characterized by elastic deformations with a nonlinear stress–strain curve. As a result, energy-based models have been explored to determine the kinematic and dynamic behaviors of these systems from this perspective [98].

**Figure 4:**
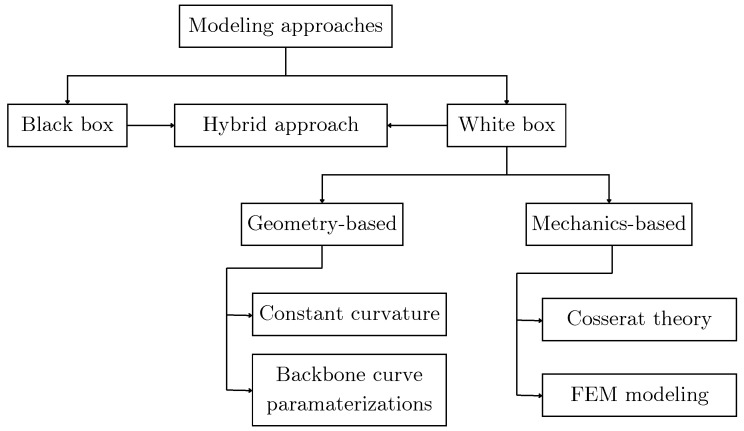
Modeling techniques for soft robotics. Adapted from [99].

The modeling of soft robotic manipulators combines comprehensive constitutive models of material deformation with the nonlinear kinematics of the manipulator itself [61]. A notable categorization of modeling techniques was proposed by Das and colleagues [99]. A summary of the proposed categorization is illustrated in Figure 4. Das and colleagues differentiate between three main categories of modeling techniques; black box models, which include models such as neural networks; white box models, which use physics-based models; and hybrid approaches that use both black and white box models.

Among the white box modeling methods, Das and colleagues differentiate between geometry-based models and mechanics-based models. Geometry-based models do not include the mechanics of the robot’s material, thus they tend to fail to represent the system when variables like gravity and payload are under consideration; some geometry-based models include Constant Curvature [100] and Backbone Curve Parametrization [101]. On the other hand, mechanics-based models allow the formulation of dynamic models, which are required for the design of control strategies for dynamic tasks. For mechanics-based models, the authors highlight the use of Cosserat Rod Theory [102]. This method allows one to model one-dimensional, slender rods by considering factors such as bending, twisting, stretching, and shearing; these models are governed by a series of nonlinear partial differential equations (PEDs), leading to complex models that are difficult to use when designing feedback controllers. Looking for simpler models, researchers have used techniques such as the Finite Element Method (FEM) [103], which is a numerical tool that gives approximate solutions to partial differential equations; these tools are useful for accurately modeling the kinematics and dynamics of a soft robot.

Table 6 summarizes different modeling approaches used in the field.

## 5. Mobile and Multi-Robot Systems Based on Soft Robotics

Soft robotics has recently gained prominence in the field of robotics, enabling the development of innovative research focused on improving and optimizing how robotic devices are constructed. This multidisciplinary field integrates knowledge from materials science, electronics, mechanical engineering, control, chemistry, physics, computer science, biology, and many other domains [104]. Considering its broad scope, it is essential to highlight the latest developments and the state of the art in these areas as they relate to soft robotics.

Soft robotics has been extensively studied for the development of mobile robots that mimic natural locomotion systems. This approach grants robots additional capabilities due to the multiple degrees of freedom provided by their soft structure and biomimetic design. Notable examples in soft mobile robotics research include the design of a six-legged insect-inspired robot enabling versatile locomotion on flat terrain [105]; a locomotion system that mimics frog movement using shape memory alloys (SMAs) fabricated through additive manufacturing [45]; a robot with pneumatic actuators replicating the movement of an earthworm [106]; a dual-anchor S-shaped crawling robot capable of traversing elevated terrains due to its unique structure [107]; and a piezoelectric curved-structure robot that is robust, capable of carrying loads, climbing slopes, and operates on as little as 8 volts [108].

Similarly, soft robotics has been applied to the design and fabrication of actuators that enhance interaction with objects during manipulation tasks. Noteworthy research includes the development of pneumatic actuators [42,109], particularly PneuNet actuators [41,110]; systems and actuators based on electroactive polymers (EAPs) [61,111]; shape memory alloy (SMA) actuators [112]; and various grippers utilizing these actuation mechanisms [113,114].

On the other hand, the fabrication and assembly of these soft robotic systems pose significant challenges due to the requirement for embedding electronic and mechanical components within a deformable structure. The assembly process must ensure proper deformation and functionality of the robot or actuator. To address these challenges, various fabrication techniques have been proposed. For instance, a prototyping technique based on polyurethane foam expansion allows for integrating electrical and actuation components within the foam [40]. Other advancements include the fabrication of high-performance electronic materials using printing and transfer methods for integration into flexible elements [54]; the development of electronic materials with molecular structures that enable extreme deformation without functional loss, referred to as “molecularly stretchable electronics” [52]; and the utilization of solid oxide present in gallium for the fabrication of soft electrodes, sensors, microcomponents for microfluidic devices, self-healing circuits, reconfigurable conductors, stretchable cables, and interconnections [49].

### 5.1. Soft Robots and Multi-Robot Systems

Soft robotics has focused on developing platforms with soft characteristics, covering aspects such as fabrication, control, integration, and operation. This research field aims to understand and expand knowledge about how soft systems can be effectively integrated into heterogeneous and homogeneous environments. This is particularly important in collaborative robotics, which is a rapidly growing area that relies on prior research as a foundation for future advancements.

MRSs are a subdiscipline of artificial intelligence and robotics, which are defined as complex systems composed of multiple independent agents. These agents act as entities within the system and have specific tasks, actions, and capabilities within a given environment [115]. MRSs can be classified into two main types:

- *Homogeneous MRS:* In these systems, all agents are identical in hardware and software. They perform the same tasks and actions and possess the same capabilities.
- *Heterogeneous MRS:* In contrast, these systems involve agents with differing hardware and software configurations, each designed for specific tasks and capabilities.

**Figure 5:**
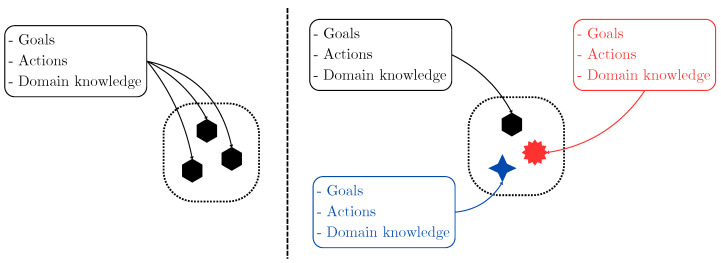
Differences between homogeneous (**left**) and heterogeneous (**right**) MRSs. Adapted from [115].

Figure 5 illustrates the fundamental differences between homogeneous and heterogeneous MRSs.

In the domain of soft robotics, one of the most notable advancements is the development of reconfigurable platforms, also referred to as Modular Self-Reconfigurable Soft Robots (MSRs). These systems are primarily oriented towards homogeneous MRSs [116]. Zhang and colleagues [117] proposed a classification of MSRs based on their functionality:

1. Assembled soft robots.
2. Reconfigurable soft robots.
3. Self-configurable soft robots.

Each category encompasses a range of research and developments, which are further detailed in the review by Zhang et al. [118].

An example of an MRS in action can be seen in scenarios where robots interact with humans, forming a two-agent system. In such cases, it is essential to analyze the tasks, actions, capabilities, and relationships among the agents to ensure effective system functionality. For instance, Ansari and colleagues developed a soft robotic manipulator to assist elderly individuals. This system utilized optimized control strategies to ensure safe and efficient interaction with the environment [104].

#### 5.1.1. Locomotion and Interaction in Multi-Robot Systems

An essential aspect of MASs and reconfigurable systems is locomotion and agent interaction mechanisms. Won and colleagues [119] developed a magneto-induced locomotion system that enhances the versatility of robotic systems and fosters magnetic interaction between soft agents. Another notable approach involves using viscoelastic polymer materials, such as hot melt adhesives (HMAs), which enable thermally regulated connection interfaces.

Table 7, adapted from Zhang and colleagues [117], provides a comparative analysis of different connection systems in reconfigurable soft robots.

#### 5.1.2. Computational Simulation in Multi-Agent Systems

Computational simulation is a critical area for the development of MASs, as it enables cost reduction during the initial prototyping stages and facilitates the prediction of agent interactions and behaviors in specific environments. By replicating complex scenarios in virtual environments, simulation allows researchers to test hypotheses, evaluate system performance, and optimize designs before physical implementation, significantly accelerating development cycles.

Austin and colleagues [120] introduced a library called Titan, built on NVIDIA’s CUDA platform, which enables the simulation of MAS environments involving soft robots. Titan’s capability to handle the unique properties of soft robotics—such as high deformability and nonlinear dynamics—makes it an invaluable tool for the design and validation of these systems. The library supports large-scale simulations with high computational efficiency, allowing researchers to explore intricate interactions between multiple soft robotic agents under varying conditions.

Furthermore, simulation tools like Titan allow for the exploration of control strategies and coordination algorithms in MASs. For example, researchers can analyze the impact of centralized versus decentralized control architectures, test communication protocols, and investigate energy consumption patterns in collaborative tasks. These simulations not only provide insights into system-level performance but also help identify potential failure modes that may not be evident in physical prototypes.

Recent advancements in computational power and the integration of machine learning techniques have further enhanced the role of simulation in MASs. Machine learning models, when combined with simulation, can predict complex behaviors and improve decision-making processes within MASs. Additionally, reinforcement learning techniques can be employed to train agents in virtual environments, enabling them to adapt to dynamic and unstructured scenarios without the risk and cost of real-world experimentation.

## 6. Key Findings

This review highlights significant advancements and challenges in soft robotics, emphasizing their integration with multi-robot collaboration. The combination of Multi-Agent Systems (MASs) and soft robotics presents great potential for collaborative applications in dynamic and unstructured environments. Applications like object manipulation, space exploration, personal assistance, and its use in medical and logistics industries, demonstrate the transformative potential of soft robotics. These findings reveal the importance of continued research to address challenges in materials, control, and integration, pushing the boundaries of adaptability, dexterity, and task performance in soft robotic systems. However, the integration of MASs, MRSs, and soft robotics faces several key obstacles. One of the primary challenges is the complexity of control and coordination, as MASs and MRSs rely on predefined kinematic models, whereas soft robots exhibit nonlinear, hyper-redundant behavior that complicates distributed decision-making and real-time adaptation. Additionally, designing safe and adaptable systems remains a critical issue, as soft robotic materials introduce compliance and flexibility that can hinder precise motion control. Scalability and communication overhead further impact large-scale coordination, given the delayed and inconsistent feedback from deformable materials.

Advances in soft actuators, including pneumatic, electroactive, thermal, and magnetic devices, have introduced versatile solutions with high flexibility, lightweight properties, and adaptability to diverse tasks. Similarly, progress in soft sensors, such as inductive, resistive, capacitive, and optical types, has enabled seamless integration of sensing capabilities into deformable structures, enhancing functionality and enabling innovative designs. However, the embedding of deformable electronics for fully autonomous systems remains a significant challenge, particularly in MAS-based frameworks requiring robust feedback mechanisms.

Fabrication techniques, including silicone elastomer molding, nanomaterial integration, conductive polymers, and liquid metals, have significantly advanced the field, allowing for the creation of highly adaptable soft robotic structures. Despite these advancements, the challenge of integrating soft materials with traditional MRS hardware and ensuring durability and repeatability in real-world applications remains unsolved. The complexities introduced by soft robotic actuation mechanisms, including hysteresis, mechanical fatigue, and nonlinear actuation responses, further hinder their widespread implementation in MAS-based robotics.

Controlling soft robots introduces further challenges due to their hyper-redundant structures and nonlinear dynamics. Solutions such as model-based control, deep learning strategies, and energy-based modeling have shown promise in improving soft robotic adaptability. Computational simulations play a pivotal role in optimizing designs and predicting dynamic interactions, with tools like the Titan library facilitating the modeling of nonlinear dynamics and large deformations. However, a lack of unified simulation frameworks that can effectively model MAS, MRS, and soft robotics interactions remains a key research gap.

Finally, energy efficiency and power management are significant concerns, as many soft robotic actuators rely on external power sources, such as pneumatic pumps or high-voltage power supplies, limiting their autonomy within MAS–MRS frameworks. Addressing these challenges requires the development of novel hybrid control architectures, enhanced multi-agent learning strategies, and improved simulation tools that account for both the distributed nature of MASs and the compliant behavior of soft robotic systems. Overcoming these obstacles will enable the next generation of adaptive, scalable, and energy-efficient robotic systems, capable of operating in complex, unstructured environments.

In Table 8, the distribution of conducted studies is presented, showing the percentage of research focused on different aspects of soft robotics. This categorization highlights the emphasis placed on sensors, actuators, materials, manufacturing methods, control strategies, and mathematical models, providing an overview of the current research landscape in the field.

The analysis of the literature highlights the distribution of research efforts across various categories in Multi-Agent Systems (MASs), Multi-Robot Systems (MRSs), and soft robotics. The most extensively studied areas include MAS coordination mechanisms and the integration of MASs, MRSs, and soft robotics, representing approximately 16.9% and 14.2% of the total studies, respectively. Research on MRS collaboration strategies accounts for 13.3% of the reviewed literature, demonstrating significant interest in enhancing teamwork among robotic agents.

Advancements in soft robotics, particularly in actuation and sensing, constitute 10.7% and 12% of the studies, respectively, reflecting ongoing efforts to develop more adaptive and responsive robotic systems. Hybrid control strategies combining MAS principles with soft robotics account for 9.8%, while fabrication techniques for soft materials represent 8.9% of the examined studies.

**Figure 6:**
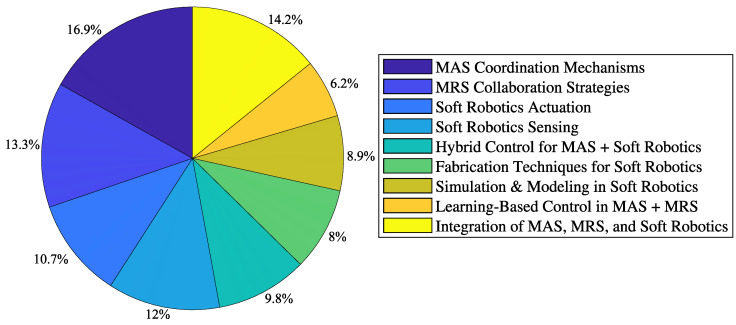
Study distribution in MASs, MRSs, and soft robotics.

Computational approaches, including simulation and modeling in soft robotics, account for 8% of the reviewed literature, highlighting the role of digital twin technologies in optimizing robotic performance. Additionally, learning-based control techniques within MAS and MRS frameworks constitute 6.2%, underscoring the growing importance of AI-driven adaptation in multi-robotic coordination. Figure 6 illustrates the proportional contributions of each research category. These findings emphasize the interdisciplinary nature of MAS, MRS, and soft robotics research and indicate key areas for future investigation.

## 7. Challenges and Future Directions

**Figure 7:**
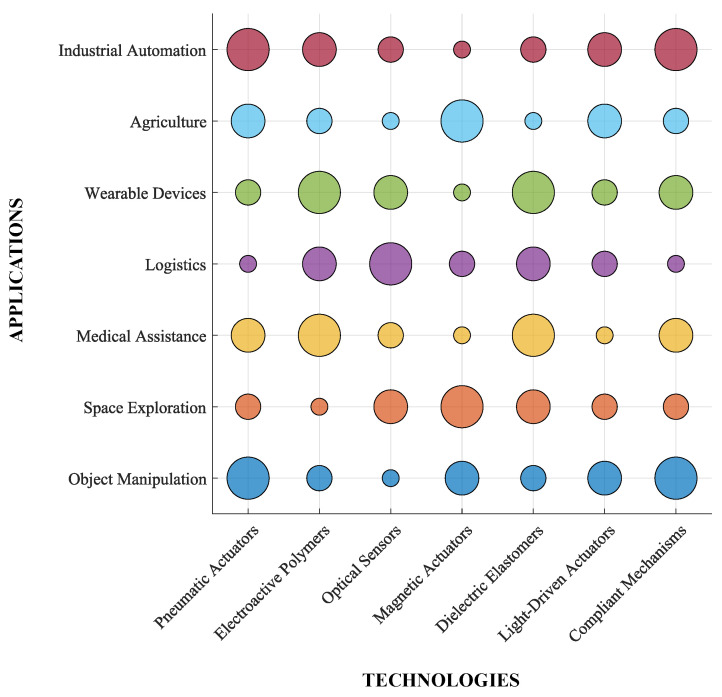
Bubble chart mapping applications vs. enabling technologies.

The integration of soft robotics presents opportunities to enhance capabilities across various domains. To visualize these advancements and their challenges, Figure 7 presents a *bubble chart* mapping applications (e.g., medical assistance, industrial automation, and wearable devices) against key enabling technologies such as pneumatic actuators, electroactive polymers, and compliant mechanisms. The size of each bubble reflects the relative suitability of a given technology for a specific application, illustrating how overlapping contributions from different technologies enable interdisciplinary approaches.

**Figure 8:**
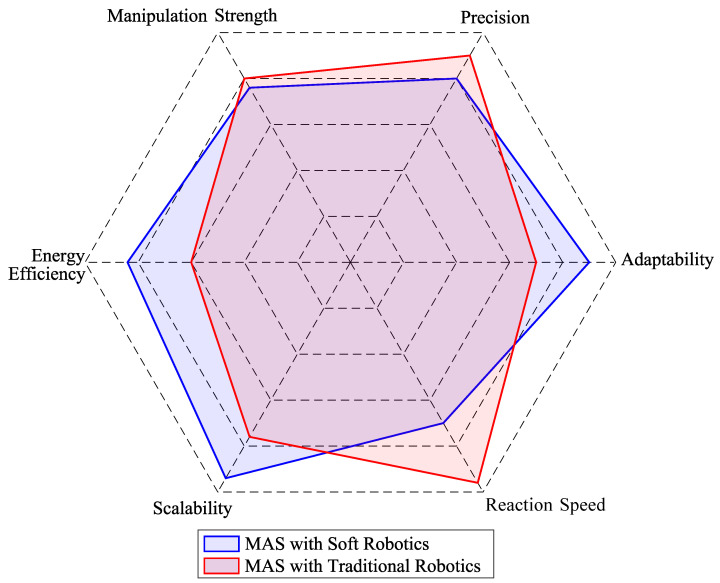
Spider plot comparing MASs with soft robotics and traditional robotics.

Furthermore, Figure 8 provides a *spider plot* comparing the core capabilities of MASs integrated with soft robotics versus MASs integrated with traditional robotics. Adaptability, precision, manipulation strength, energy efficiency, scalability, and reaction speed are evaluated to highlight their strengths and weaknesses. The results show that MASs integrated with soft robotics excel in adaptability and scalability, offering enhanced flexibility and the ability to address complex, dynamic environments. However, MASs integrated with traditional robotics demonstrate higher precision and slightly better energy efficiency, making them more suitable for tasks requiring exactness and lower power consumption. Additionally, traditional robotics exhibits a significant advantage in reaction speed due to the rigid nature of its components and the direct transmission of forces, whereas soft robotics experiences a delay in response time due to material deformations and the inherent compliance of its structures.

These visual analyses highlight key challenges, such as improving energy efficiency in soft robotic systems, enhancing scalability in traditional paradigms, and addressing the precision gap in MASs. Table 9 summarizes the key challenges and opportunities identified in developing Multi-Robot Systems and soft robotics. These areas of opportunity highlight the integration of MASs with soft robotics, the design of advanced control strategies, innovations in fabrication and materials, computational simulation for optimization, and the expansion of collaborative applications.

Future work will explore integrating the technologies studied in this review with the design of MASs to enhance their capabilities in dynamic and complex environments. Specifically, efforts will focus on seamless hardware and software framework integration to enable effective coordination and adaptability in MASs using soft robotics. Additionally, future research will address control strategies tailored to the unique characteristics of soft robotic systems, such as nonlinear dynamics and hyper-redundant configurations. Another key area of investigation will involve developing and optimizing advanced connection mechanisms, including attachment and assembly elements, to improve the modularity and functionality of MASs integrated with soft robotics. These initiatives will provide new insights and solutions for building scalable, adaptive, and collaborative robotic systems across diverse applications.

In Table 10, the distribution of challenges, opportunities, and future directions in the integration of Multi-Agent Systems (MASs) and soft robotics is presented. This classification identifies key aspects that require further research and development, including the adaptation of control frameworks, scalability, and multi-modal sensor fusion. The table provides a structured overview of the main research gaps and emerging possibilities in this interdisciplinary field.

## 8. Conclusions

This review highlights significant advancements and challenges in integrating Multi-Robot Systems (MRSs) and soft robotics. By analyzing the state of the art in MRS coordination, soft robotic design, fabrication techniques, control strategies, and computational simulations, this work identifies key obstacles and opportunities for innovation in these fields. The integration of soft robotics into MRSs offers unique advantages, such as flexibility, adaptability, and safety in human–robot and environment–robot interactions, which are particularly valuable for systems operating in dynamic and unstructured environments.

The combination of the adaptive properties of soft robots with the coordination mechanisms of MRSs has the potential to achieve unprecedented performance levels in collaborative tasks, including object manipulation, locomotion, and environmental interaction. Advances in soft actuation technologies, including pneumatic, electroactive, and magnetic actuators, have improved compliance and dexterity. Meanwhile, deformable sensors, such as inductive, resistive, and capacitive types, have enhanced real-time feedback, enabling precise interaction with the environment. Fabrication techniques, including silicone molding, conductive polymers, and liquid metals, have facilitated sophisticated soft robotic designs, yet the seamless integration of deformable electronics remains a challenge.

Despite these advancements, several obstacles persist. Developing robust control algorithms capable of handling the nonlinear, hyper-redundant properties of soft robots is essential for their efficient integration into MAS-based frameworks. Additionally, ensuring the scalability of multi-agent coordination, improving energy efficiency, and designing advanced simulation tools capable of modeling both discrete and continuous system dynamics are critical areas for future research. Exploring novel materials such as hydrogels, liquid metals, and nanomaterials presents further opportunities to enhance the functionality and efficiency of soft robotic systems.

Future work should focus on hybrid control architectures, improved computational models for MAS–soft robotics interaction, and reinforcement learning-based strategies for autonomous adaptation. Overcoming these challenges will drive the development of next-generation robotic systems capable of seamless cooperation, high adaptability, and efficient operation across diverse real-world applications.

While this review provides a comprehensive analysis of the integration of MASs, MRSs, and soft robotics, certain limitations must be acknowledged. First, the scope of this study is restricted to peer-reviewed articles available in databases such as SCOPUS and Web of Science, which may exclude relevant contributions from emerging research repositories, technical reports, and industry developments. Additionally, the rapid advancements in soft robotics and MASs introduce a challenge in maintaining an up-to-date synthesis, as new technologies and methodologies continue to evolve beyond the timeline of this review. Finally, despite identifying key challenges in MASs and soft robotics integration, this review does not extensively address ethical, economic, or deployment considerations that could impact large-scale adoption in industrial or biomedical applications. Future research should aim to incorporate experimental validation, cross-disciplinary evaluations, and broader technological forecasting to strengthen the applicability and impact of MAS-driven soft robotic systems.

The future of robotics lies in the seamless integration of MRSs and soft robotics. Addressing the outlined challenges will enable breakthroughs in sectors such as healthcare, logistics, space exploration, and personal assistance.

## Figures and Tables

**Table 1:** Fabrication Methods in Soft Robotics.

Method	Description	Applications
Injection Molding	Polymer molding for flexible parts	Soft component fabrication
3D Printing	Additive manufacturing of soft structures	Rapid prototyping
Photopolymerization	UV light-curing materials	Microactuators
Lithography Assembly	Layered microstructure fabrication	Soft electronics
Laminated Layering	Stacked multimaterial components	Hybrid materials

**Table 2:** Materials Used in Soft Robotics.

Material Type	Properties	Applications
Silicone	Elasticity, biocompatibility	Medical robots, flexible sensors
Hydrogels	Water absorption and contraction	Biomedical actuators
Shape Memory Alloys	Thermal shape recovery	Microactuators, prosthetics
Conductive Elastomers	Flexible conductivity	Flexible sensors and control systems
Magnetic Materials	Magnetic responsiveness	Contactless actuators

**Table 3:** Types of Actuators in Soft Robotics.

Actuator Type	Mechanism	Applications
Pneumatic	Air chamber expansion	Soft object manipulation
Electroactive Polymers	Structural change under electric field	Fast actuation in biomimetic devices
Magnetic	Rigidity modification under magnetic field	Medical and industrial applications
Dielectric Elastomers	Electrostatic contraction	High-energy efficiency robots
Light-Driven	Photoactuated contraction	Microrobots

**Table 4:** Types of Sensors in Soft Robotics.

Sensor Type	Function	Applications
Tactile	Pressure and deformation	Contact detection, object manipulation
Deformation	Elongation measurement	Motion monitoring
Optical	Light intensity modification	Position sensing in soft robots
Magnetic	Magnetic field detection	Direction and force control
Temperature	Thermal sensitivity	Thermal regulation

**Table 5:** Control Strategies in Soft Robotics.

Control Strategy	Principle	Applications
Model-Based Control	Dynamic modeling with physics equations	Predefined geometry robots
Learning-Based Control	Neural networks for adaptation	Autonomous robots
Sliding Mode Control	Nonlinear system adjustments	High-variability systems
Sensor Feedback	Real-time movement correction	Precision manipulators

**Table 6:** Mathematical Models in Soft Robotics.

Model Type	Principle	Applications
Black Box Models	Neural networks, supervised learning	Data-driven robots
Geometric Models	Curvature and deformation approximations	Soft structure kinematics
Mechanics-Based Models	Force, tension, and material considerations	Precision robot control
Hybrid Models	Physical models and neural networks	Autonomous adaptive systems

**Table 7:** Connection Mechanisms in Modular Self-Reconfigurable Soft Robots (MSRs). Adapted from [117].

Connection Mechanism		Advantages	Disadvantages
Mechanical	Rigid connector	High connection strength	Add complexity to the modular units
		Good alignment precision	Add rigidity to soft modular units
	Soft connector	Good compliance	Low connection strength
Magnetic	Permanent Magnet	Easy attachment	Need extra actuation for disconnection
		High connection strength	Add rigidity to soft modular units
		Self-aligning	
	Electromagnet	Easy connection and disconnection	Need an extra power source
		High connection strength	Adding complexity to modular units
		Self-aligning	Add rigidity to soft modular units
Adhesive	Glued	High connection strength	One-off and irreversible
		Low design requirements	
	Holt-melt	Easy connection and disconnection	Need an extra heating strategy
		Compact design	
	Electrostatic	Not require precise alignment	Need an extra power source
		Easy connection and disconnection	Low connection strength
Vacuum	Sucker	Easy connection and disconnection	Depend on precise alignment
		High connection strength	Require an integrated pump actuation system

**Table 8:** Distribution of Studies in Soft Robotics.

Category	Percentage (%)
Sensors	22.73%
Actuators	16.36%
Materials	20.00%
Manufacturing Methods	13.64%
Control Strategies	18.18%
Mathematical Models	10.91%

**Table 9:** Challenges and Opportunities in Multi-Robot Systems and Soft Robotics.

Area of Opportunity	Challenges	Opportunities
Integration of MASs with soft robotics in dynamic environments	MASs face difficulties in coordinating heterogeneous agents in dynamic environments, especially when integrating soft robots with unique properties such as flexibility and adaptability.	Design collaborative systems that leverage the features of soft robots, such as safety in interaction and adaptability to various tasks, in applications where traditional MRSs have limitations.
Design of advanced control strategies	Soft robots present modeling and control challenges due to their nonlinear properties and hyper-redundant degrees of freedom.	Develop intelligent control algorithms based on deep learning and predictive models to manage the kinematic and dynamic complexity of these systems.
Innovations in fabrication and materials	Incorporating deformable electronic components and improving assembly processes to ensure functionality and durability in soft structures.	Explore advanced materials such as hydrogels, liquid metals, and nanomaterials that offer adaptive properties and enhance the capabilities of soft robots.
Computational simulation applied to MRSs with soft robots	Simulating complex environments that replicate physical interactions and behaviors of Multi-Robot Systems with soft robots.	Use tools like the Titan library based on CUDA to optimize the design and performance of these systems before physical implementation.
Expansion of collaborative applications	Ensuring compatibility and synchronization between heterogeneous robots in collaborative tasks.	Implement MASs combined with soft robotics in sectors such as healthcare, logistics, space exploration, and personal assistance, taking advantage of their adaptability and ability to interact in unstructured environments.

**Table 10:** Challenges and Opportunities in MASs and Soft Robotics Integration.

Challenge/Opportunity	Category	Impact
Nonlinearity and hyper-redundant dynamics	Challenge	High
Control framework adaptation	Challenge	Medium
Lack of standardized simulations	Challenge	High
Advanced control strategies	Opportunity	High
Hardware–software co-design	Opportunity	High
Collaborative object manipulation	Opportunity	Medium
Scalability and distributed intelligence	Opportunity	High
Bio-inspired MAS approaches	Future Direction	Medium
Multi-modal sensor fusion	Future Direction	High
Standardization of benchmarking metrics	Future Direction	Medium

## Data Availability

The original contributions presented in the study are included in the article; further inquiries can be directed to the corresponding author.
